# ReviewGenie: a novel automated system for systematic reviews—an exploratory study in speech and language disorders

**DOI:** 10.1186/s13643-025-02895-z

**Published:** 2025-08-18

**Authors:** Abeer Z. Al-Marridi, Ahmed Bensaid, Samawiyah M. Ulde, Tariq Khwaileh

**Affiliations:** 1 Department of English Literature and Linguistics, Qatar University, Doha, 2713 Qatar; 2 Data Analytics and Assessment Section, Qatar University, Doha, Qatar

**Keywords:** Automatic, Screening, Systematic review, LLM, ReviewGenie, Speech and language disorders

## Abstract

**Background:**

Systematic reviews (SRs) are a cornerstone in providing high-quality evidence that guides policy and practice across various disciplines. Despite their critical role, SRs require substantial financial investment and are constrained by time-consuming manual processes. Existing solutions primarily focus on semi-automating the title and abstract screening stages, yet these approaches still face limitations in terms of efficiency and practicality. The SR process comprises several stages beyond abstract screening, each of which is resource-intensive. To overcome these challenges, this paper introduces ReviewGenie, a novel system that automates SR stages up to and including abstract screening, utilizing artificial intelligence.

**Method:**

The SR process involves eight key stages, beginning with the definition of search keywords and the selection of target databases, and culminating in full screening. While the initial and final stages require human expertise, the intermediate stages can be automated. ReviewGenie automates all intermediary stages, including database searching, data retrieval, cleaning, deduplication, filtering, and abstract screening. The system is domain-agnostic, as evidenced by a case study focused on databases related to speech and language disorders.

**Results:**

ReviewGenie significantly reduces the workload across various stages of the SR process, delivering notable time and cost savings while enhancing efficiency and accuracy. In the case study, where the article-fetching stage involved tens of thousands of publications, ReviewGenie achieved a 2.62% improvement in duplicate detection in less than a second, compared to the 1 to 3 h typically required for manual deduplication of 100 records. This process included cleaning abstracts before removing duplicates. Additionally, ReviewGenie reduced the number of articles from 28,674 to 3520 using an automatic filtering approach executed in seconds. This substantial reduction underscores the effectiveness of our automated method in preparing datasets for the abstract screening stage. Moreover, the artificial intelligence-driven abstract screening method resulted in cost savings exceeding $6230 compared to manual methods.

**Conclusions:**

ReviewGenie represents a significant advancement in reducing the burden on researchers conducting comprehensive systematic reviews. By automating intermediate stages, ReviewGenie enhances efficiency, accuracy, and cost-effectiveness, establishing itself as an indispensable tool for SRs across various disciplines.

## Background

Systematic reviews (SR) are comprehensive analyses of research studies that adhere to a structured and transparent process to synthesize all relevant evidence. SRs establish a reliable foundation for assessing the effectiveness of interventions. The growing volume of research contributions has raised the importance of SRs in providing evidence-based insights for decision-making and advancing the frontier of scientific knowledge. However, the increasing complexity of research and the rapid pace of publication have transformed SRs into resource-intensive undertakings. The traditional manual process, often requiring multiple researchers and spanning several years to complete, faces challenges from rapidly aging evidence. To mitigate this, the Cochrane Handbook for Systematic Reviews recommends conducting final searches within 6 months of publication to maintain the relevance of the review [1]. The associated costs can exceed $100,000, accounting for the time invested in searching (conducted by information specialists), screening, extracting data, analyzing, interpreting, and writing performed by methodologists [2]. For over a decade, significant efforts have been made to automate SR tasks. Advances in technology, computational power, and artificial intelligence (AI) have increasingly enabled the integration of these innovations into the SR process, aiming to accelerate workflows, enhance efficiency, and improve accuracy. Researchers have explored automation across various stages of the SR process, both broadly and within specific domains [3]. Covidence, a well-established cloud-based systematic review tool, does not feature machine learning-assisted (ML) abstract screening but provides other enhancements such as team and project management, and conflict resolution for disagreements [4].

Conversely, ML algorithms hold promise for automating the screening process. By learning from human-labeled data, ML algorithms can classify articles as relevant or irrelevant, potentially reducing the time and resources required for SRs [5]. In [6], the authors introduced GAPscreener, a support vector machine based approach designed to streamline the screening of human genetic association studies in PubMed, aiming to improve efficiency and accuracy. However, the system’s performance is limited by the quality and scope of the input data. Additionally, GAPscreener’s utility is confined mainly to genetic association research, often requiring manual verification to ensure reliability. The tool also demands regular updates to stay current with new studies, adding to the burden on reviewers and developers.

In [7], the authors compared three prominent screening tools: Abstrackr, DistillerSR, and RobotAnalyst. All of these tools necessitate the availability and upload of screened documents into their systems. The study concluded that Abstrackr demonstrated the best performance among the three, while DistillerSR and RobotAnalyst showed varying levels of success. Despite their advantages in enhancing efficiency, all these tools remain semi-automated and still necessitate human involvement, along with high risk of missing relevant studies. The initial phase of screening, which is crucial for training their models, must be carried out manually. This process continues until a sufficient number of relevant articles have been identified to effectively train a text classifier. Although the exact number of positive examples required for optimal predictive performance can vary, a conservative estimate is to use about half of the retrieved articles. Once trained, the classifier predicts the relevance of all unscreened abstracts, which are then reordered based on their predicted relevance. Tools such as EPPI Reviewer [8], SWIFT-Review [9], Colandr [10], and Rayyan [11] demonstrate this approach as well. While these systems save time compared to traditional methods, they often miss relevant records and still require manual oversight for uploading and initial screening [12].

A notable challenge in automated abstract screening is determining when it is “safe” to stop manual review, which varies by review. Automated systems generally rank articles by relevance likelihood rather than providing definitive classifications [13]. Most of the approaches mentioned fall under the active learning paradigm in ML, where the model learns from informative data points (e.g., selected articles) rather than using random samples. This method, while efficient, introduces the potential for bias, as the results can be influenced by the criteria used to select the data points. To address this, the authors in [14] proposed a “SAFE procedure” to balance the costs of continued screening against the risk of missing relevant records. However, this approach requires careful tuning of parameters specific to each systematic review, considering the number of records to screen, the minimum percentage of records to check, and the threshold for consecutive irrelevant records. Poorly adjusted parameters could compromise the accuracy of the results. It is worth noting that most of the tools discussed primarily serve to enhance the transparency of the screening process by facilitating communication among reviewers [15]. Therefore, it would be more accurate to refer to them as review management software rather than screening tools. In [16], the authors proposed a semi-automated abstract screening tool that surpasses others but has several limitations. It requires full-text article access and constant researcher oversight. Even with ML-based ranking, researchers must flag each article manually through up to 50 iterations. The tool’s accuracy is also highly dependent on the proper setting of seed articles.

The authors in [17] introduced an enhanced version of the BERT model[18], called srBERT, specifically designed to classify articles based on their abstracts. However, the effectiveness of srBERT is significantly influenced by the size and quality of the training data. Securing a high-quality dataset, particularly in specialized fields, remains a challenge and can limit the model’s performance. Retraining BERT on a specific corpus has been proposed by some researchers to leverage its capabilities in data extraction and classification; however, they continue to face the aforementioned limitations [19, 20]. While screening tools driven by computing, software development, and ML have shown promising results, they still possess limitations that need to be addressed, providing opportunities for further innovation. The research community found that different tools have varying accuracy across different reports [21]. Current tools for SRs primarily focus on document screening and require substantial data input, often necessitating reviewers to screen hundreds or thousands of articles to train the model. This limits time savings, rendering such tools inefficient for small-scale SRs and still burdensome for large ones. While some tools support de-duplication, their accuracy is limited due to the lack of abstract cleaning prior to this step.

Recent advancements in large language models (LLMs) have showcased their remarkable ability to handle a diverse range of tasks across various domains. Zero-shot learning, which involves tackling tasks without specific training data, poses a significant challenge for these models, which usually depend on extensive datasets. The capability to perform new tasks based solely on given instructions marks a critical milestone toward achieving artificial general intelligence, as highlighted in [22].

The first notable LLM to demonstrate zero-shot learning was OpenAI’s GPT-2, released in 2019 [23]. Pre-trained on over 8 million documents (40 GB of text), GPT-2 generated coherent text across various topics without task-specific fine-tuning. GPT-3 followed in 2020, with 175 billion parameters and a pre-training dataset of over 45 TB. In 2023, GPT-4 was released with further architectural advancements [24].

According to [25], GPT has outperformed other methods in few-shot learning across various tasks. Recently, AI models like Microsoft Bing, and Google Gemini have been developed for zero-shot tasks, achieving success in domains such as law, business, education, and medicine. In the medical field, despite concerns about AI adoption, these models have been applied to decision-making, patient education, drug-drug interaction identification, physician-patient communication, and clinical outcome prediction [24, 26–28]. In [24], the authors investigated GPT-3.5’s accuracy in neurolocalization, achieving an accuracy score of 84.8%, indicating promise but not yet reliability for routine patient care. Another study [26] assessed the sensitivity, specificity, and accuracy of GPT-3.5, GPT-4, Bing AI, and Bard (Google Gemini) in predicting drug-drug interactions, with GPT-4 demonstrating superior sensitivity in identifying true positive drug-drug interactions. The authors in [29] compared GPT-3.5, Microsoft Bing, and Google Gemini for diagnosing neuro-ophthalmological cases, concluding that GPT-3.5 outperformed Bing and Gemini, providing accurate responses in 60% of cases, while the others achieved accuracy rates of 50%. All three models correctly diagnosed 40% of the cases. In [30], a study evaluating the clinical accuracy of GPT-3.5, GPT-4, and Llama 2 models compared to Google search for diagnosing, examining, and treating 110 medical cases, GPT-4 outperformed the others, especially in diagnosis and examination, while Llama 2 showed slightly lower performance. In [31], the authors study the effectiveness of using the GPT model to extract useful records from complex scientific knowledge. The authors in [32, 33] supported using LLMs for title and abstract screening in SRs. Their research demonstrated that publicly available LLMs yielded promising results, achieving acceptable levels of accuracy. Since 2022, LLMs have rapidly gained prominence as the fastest-growing consumer software applications [26, 27]. Among them, GPT has demonstrated strong alignment with human judgements and significant academic impact, recognized by Nature in 2023 as a top researcher and frequently appearing as a co-author [34].

Despite comprehensive literature reviews, no fully automated system has yet been developed to streamline the entire systematic review process, from article retrieval to abstract screening. Existing solutions invariably require significant human intervention.

Our comprehensive review of literature on automating systematic reviews (SRs) from 2014 to 2024, encompassing title-abstract screening and deduplication, reveals that existing systems fall short of achieving complete automation. Key limitations include (1) heavy reliance on manual intervention for model training and data management, (2) dependence on high-quality, comprehensive datasets that are often difficult to obtain, (3) burden on reviewers imposed by the requirement to upload full-text articles, especially for those with limited access to e-databases, and (4) potential for missing relevant studies due to incomplete automation and challenges in determining when to cease manual review.

**Fig. 1:**
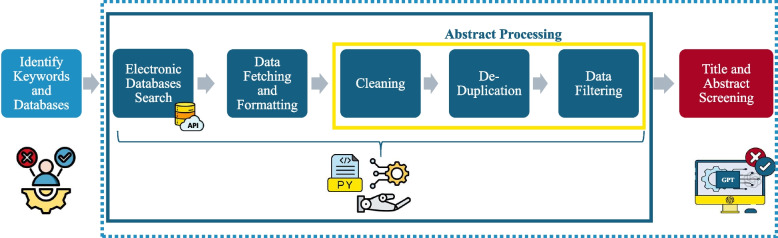
Automated systematic review workflow. Keyword and database selection is the only step requiring human input. Processes within the dashed box are fully automated, using Python scripts for initial processing and formatting, and a LLM for final decision-making. Steps within the yellow box represent pre-processing and filtering of retrieved abstracts before submission to the AI model

To address these challenges, we developed and refined ReviewGenie, a fully automated review system that covers the SR process till screening titles and abstracts based on predefined inclusion and exclusion criteria. The system is designed as a structured, seven-step process to enhance the efficiency, consistency, and accuracy of literature retrieval and analysis. It begins with identifying keywords and selecting appropriate databases, followed by automated searches and data fetching from these sources. The process also includes data cleaning, de-duplication, and filtering to refine the dataset to the most relevant studies. Finally, the system utilizes GPT for title and abstract screening, ensuring a streamlined and accurate review process. Each stage is meticulously designed to address specific challenges and improve overall performance, offering significant time savings and consistency in systematic reviews, as illustrated in Fig. 1. The paper aims to address the following research questions.

- How does ReviewGenie compare to traditional manual methods in terms of accuracy, completeness, and filtering of retrieved articles?
- What is the quantitative impact of ReviewGenie on time and cost efficiency across all stages of systematic reviews, compared to semi-automated and manual approaches?
- How effectively does ReviewGenie handle large-scale systematic reviews, and what are its limitations?

## Methods

ReviewGenie is organized into a seven-step process, with each stage designed to tackle specific challenges and improve overall performance.

### Identify keywords and databases

#### **Stage description:**

the first step involves deciding on the general and specific search queries relevant to the research topic. Once the keywords are established, appropriate electronic databases are selected to retrieve relevant literature. This initial stage is crucial for setting the parameters that guide the subsequent stages of the automation workflow.

#### Implementation:

- Keywords: define broad and narrow keywords that capture the essence of the research topic.
- Databases: select digital library databases such as IEEE Xplore, PubMed, Embase, PsycINFO, among others, based on the scope and nature of the research.

### Automatic electronic databases search

#### Stage description:

After selecting the relevant databases and keywords, an advanced search query is constructed and executed using the database’s API to automate the retrieval of relevant literature. For databases with direct API access, such as PubMed and IEEE Xplore, this process is fully automated. However, for databases that lack public API access, such as Embase and APA PsycInfo, data retrieval was performed manually by exporting the relevant results in Excel sheet format, making them eligible for the next stage of the process or allowing for the use of alternative third-party tools.

#### Implementation:

Utilizes the APIs provided by the selected databases and formulates advanced search queries using Boolean operators and database-specific syntax to ensure comprehensive search results. Algorithm 1 provides an overview of the process for retrieving articles from electronic databases (e-databases) based on specific queries. While the algorithm is shown for a single query for simplicity, it can be extended to handle multiple queries in parallel. Multiple predefined queries can be executed in a loop, with results concatenated before saving to an Excel sheet.

### Automatic data fetching of the returned files

#### Stage description:

Search results from multiple databases are extracted, including metadata such as title, abstract, publication date, DOI, publisher name, and citation count. These results are subsequently aggregated into a single Excel spreadsheet to maintain uniformity across all entries. The information is meticulously selected based on the research objectives and in accordance with predefined inclusion and exclusion criteria.

#### Implementation:

- Data fetching: retrieving data from e-databases and storing it in a structured format can be challenging, especially when some databases, such as Embase, do not support direct export to Excel files or have limited API access. In such cases, a programming approach is necessary. This involves extracting data from exported text files using specific keywords and then organizing the data into an Excel file. Different scripts may be required depending on the database and its export capabilities. Algorithm 2 illustrates the procedure for extracting data from exported Embase unstructured text files and organizing it into a structured Excel format. This process can be repeated for all text files corresponding to multiple queries by using a loop, as needed by the researcher. Data fetching: retrieving data from e-databases and storing it in a structured format can be challenging, especially when some databases, such as Embase, do not support direct export to Excel files or have limited API access. In such cases, a programming approach is necessary. This involves extracting data from exported text files using specific keywords and then organizing the data into an Excel file. Different scripts may be required depending on the database and its export capabilities. Algorithm 2 illustrates the procedure for extracting data from exported Embase unstructured text files and organizing it into a structured Excel format. This process can be repeated for all text files corresponding to multiple queries by using a loop, as needed by the researcher.
- Data formatting: merge results from different databases into a single Excel sheet, a systematic approach is required. This involves automatically consolidating data from various sources using the Pandas library in Python. The resulting Excel sheet includes articles from all searched e-databases with headers: Title, Abstract, Publication Date, DOI, and Database. Researchers may add columns like the number of citations based on their criteria, which should be considered in the data fetching stage, as shown in Algorithm 2.

**Figure:**
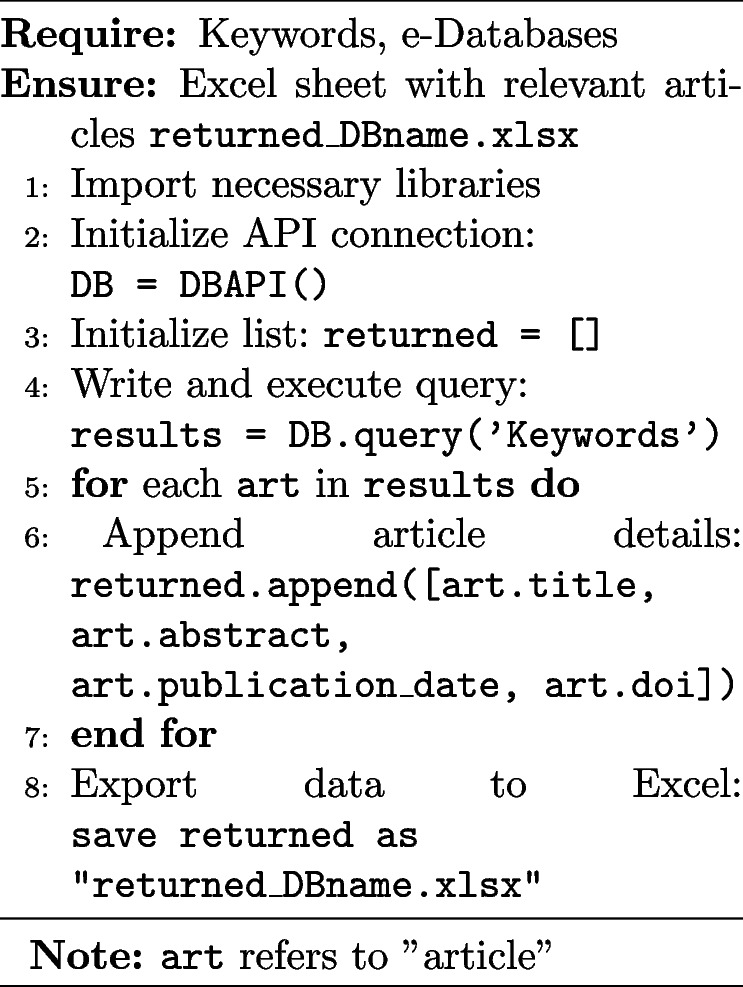
**Algorithm 1** Retrieve articles from e-databases

**Figure:**
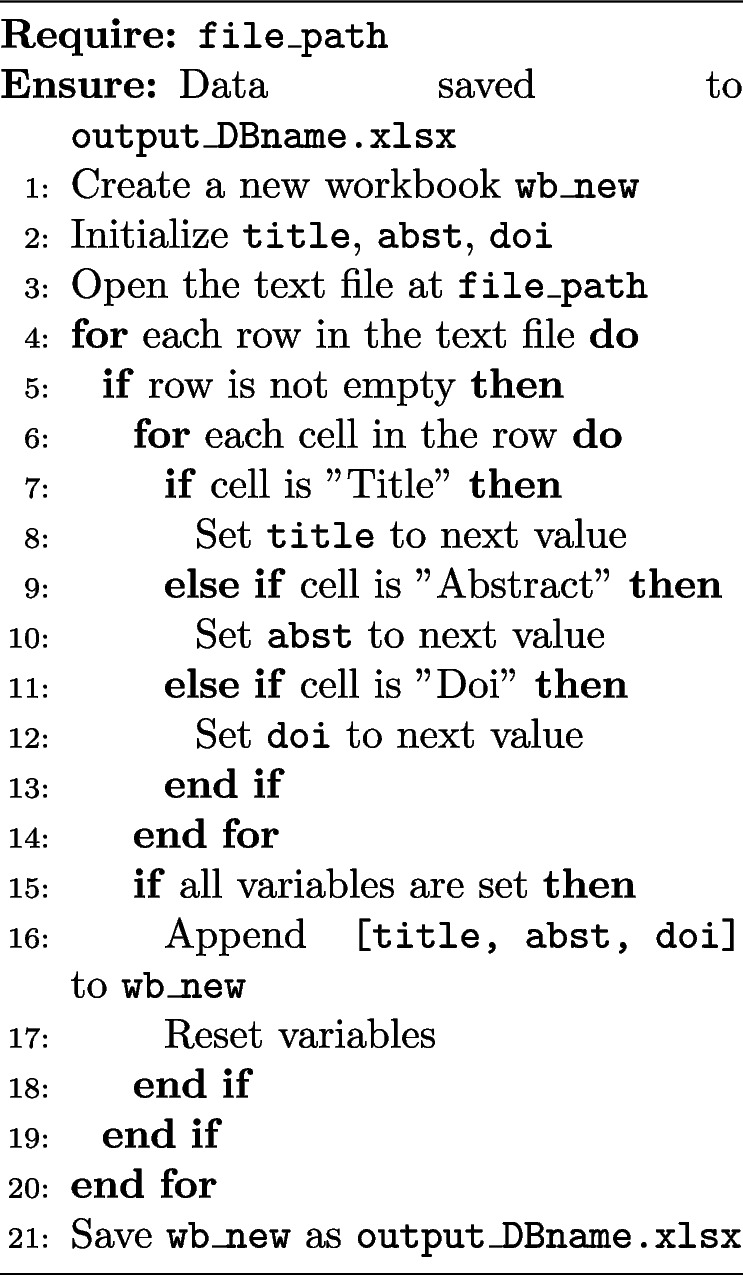
**Algorithm 2** Extract and combine data from text files

### Automatic cleaning of the returned abstracts

#### **Stage description:**

Clean the abstracts by removing punctuation and other non-essential characters, ensuring the data is ready for further processing.

#### Implementation:

Cleaning and preprocessing the abstracts by applying text processing techniques to remove punctuation and irrelevant characters. Define a Python function using str.maketrans and string.punctuation to create a translator that replaces punctuation and irrelevant characters with spaces, effectively removing them from the text.

### Automatic de-duplication of all results

#### Step description:

Ensure uniqueness in the dataset, duplicates across the collected data are identified and removed.

#### Implementation:

De-duplication: utilize Python text processing libraries (i.e., Pandas) to detect and eliminate duplicate entries based on specific criteria such as title and abstract content. Automating the de-duplication process significantly enhances the efficiency of the systematic review process. Traditionally, manual de-duplication can vary widely in time, influenced by factors such as the volume of records, data complexity, and reviewer expertise. On average, this task can take between 1 and 3 h per 100 records [35]. In contrast, our automated implementation completes de-duplication in less than a second, demonstrating a substantial reduction in processing time and highlighting the value of automation in streamlining systematic reviews.

### Automatic filtering

#### Stage description:

Filter the collected literature by discarding titles and abstracts that do not contain predefined keywords, refining the dataset to the most relevant studies.

#### Implementation:

Filtering mechanisms that retain only those entries containing specific keywords within their titles and abstracts. This stage can be iterative, allowing multiple levels of filtering based on evolving research needs and insights. Algorithm 3 outlines the high-level description of filtering stage of the automation process. In this context, defining the keywords for filtering corresponds to stage one of the process.

**Figure:**
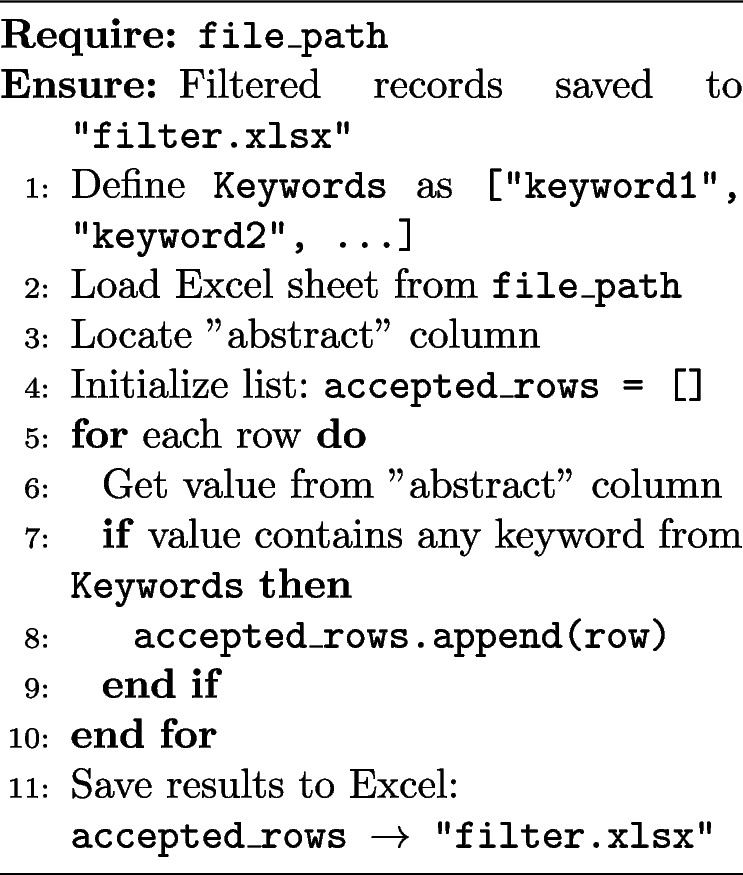
**Algorithm 3** Filter records based on keywords

### Automatic title and abstract screening

#### Stage description:

Utilize the advanced capabilities of the pre-trained LLM model to screen titles and abstracts and determine their relevance.

#### Implementation:

Create API queries for LLM (i.e., GPT) by combining the following elements: specific instructions, the publication’s title, its abstract, and predefined relevance criteria (inclusion and exclusion criteria). The model assesses a publication’s suitability for a specific research purpose based on these components. The instructions outline the task of determining a publication’s relevance using a word-based or numerical scale. This decision is made by evaluating the title, abstract, and predefined criteria. The instruction should ensure clear responses, such as a final decision expressed as a single word: “yes,” “no,” or “maybe.” These criteria, set by researchers, help identify relevant publications for a specific research topic and remain consistent across all evaluations. This approach enables an efficient and streamlined abstract screening process, with GPT demonstrating high accuracy and providing significant advantages.

## Results

Current systems require human involvement for abstract screening, whereas ReviewGenie automates the process up to that stage. However, initial setup (keyword selection, database choice, inclusion/exclusion criteria) still demands researcher expertise. To evaluate ReviewGenie’s efficacy, we conducted a comprehensive systematic review of speech and language disorders, exploring all relevant public and private databases. This research is crucial for advancing the field by offering enhanced methodologies and insights based on existing data. The following section presents ReviewGenie’s performance in this context.

### Identify keywords and databases:

A comprehensive two-tiered search strategy was implemented to identify relevant literature on speech and language disorders. This strategy utilized both general and disorder-specific keywords to ensure extensive coverage. The general keywords included Speech Disorder, Language Disorder, Corpus, Database, and Corpora, while the disorder-specific keywords encompassed fourteen distinct types of speech and language disorders. The search was conducted across the following e-databases: IEEE, PubMed, Embase, and PsycINFO, selected for their comprehensive coverage of relevant literature in engineering, biomedical research, clinical studies, and psychology.

### Automatic electronic databases search and data organization:

**Fig. 2:**
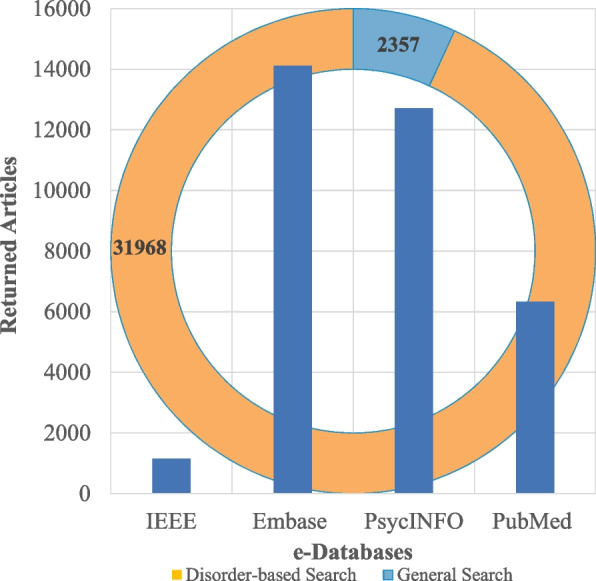
Combined graph showing the number of retrieved articles, categorized by electronic database and two-tier search strategy

Utilizing the selected keywords, each database was accessed through its API via a Python script. The script formulated advanced search queries by combining the specified keywords with search constraints, such as language and publication year range. This automation streamlined the search process, eliminating the need for manual queries and efficiently managing the retrieval of results. It is important to note that some APIs impose a daily limit on the number of search results returned. This two-stage search process resulted in more than 100 queries, identifying a total of 34,325 articles across the aforementioned databases, as illustrated in Fig. 2.

The data returned by the APIs varied in format; thus, an automated process was implemented to organize all retrieved data into an Excel spreadsheet. This process concentrated on extracting specific fields, including title, abstract, publication date, article type, and DOI. Table 1 compares the time required to process 1,000 documents manually by a single researcher with the time taken by the proposed automated approach.

**Table 1:** Time required to fetch and organize data from various files into a formatted file

File Type	Data fetching time
Excel file	12,500 s $\approx$ 3.5 h
Unstructured text	61,200 s $\approx$ 17 h
Structured text	36,000 s $\approx$ 10 h
Automatic	4 s

### Automatic abstract cleaning, de-duplication, and filtering:

**Fig. 3:**
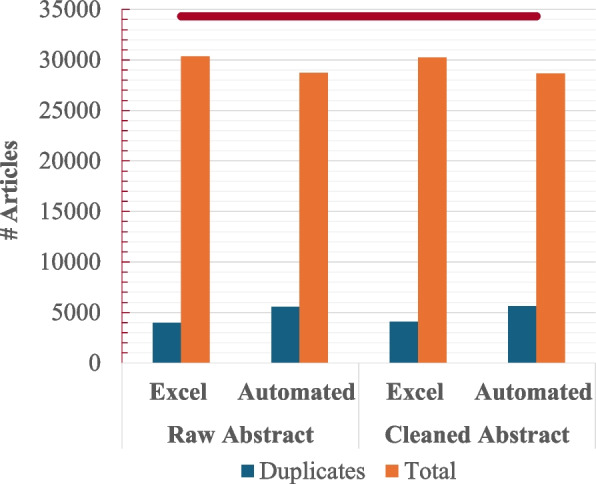
Comparison of de-duplication results for raw and cleaned abstracts using Microsoft Excel and the proposed automated approach. The red bar represents the total number of articles before de-duplication

To ensure seamless abstract screening, it is essential to perform preprocessing steps, including the de-duplication of records. We utilized Microsoft Excel’s advanced capabilities to evaluate the de-duplication of 34,325 articles against our automated approach. As illustrated in Fig. 3, our method demonstrates notable efficiency and underscores the importance of data cleaning in both approaches. De-duplication was performed on both raw and cleaned abstracts. On average, cleaning the abstracts of 34,325 articles took 4.68 s, while saving the results to an Excel sheet required 5.36 s. Thus, the combined average time for these tasks was approximately 10 s. The subsequent filtering of cleaned data took only 2 s, significantly reducing the number of articles from 28,674 to 3520. This considerable reduction demonstrates the effectiveness of our automated approach in streamlining the dataset for the subsequent abstract screening stage.

### Automatic title and abstract screening:

As highlighted by recent studies [1, 16], abstract screening is a labor-intensive process, with each review taking between 1 and 6 min depending on the abstract’s complexity and length. In our study, two reviewers (AZA and SMU) screened 1027 abstracts out of 3520 articles as part of an initial testing phase, averaging 3.5 min per record. This process required approximately 17.12 working days, assuming a 7-h workday. Inter-rater reliability was assessed using Cohen’s Kappa ($\kappa$ = 0.891), which, according to the classification proposed by [36], reflects “almost perfect agreement”. Discrepancies were resolved through consensus or, when necessary, involving a third reviewer. In contrast, the automated method completed the same task in approximately 11.37 min.

Table 2 presents a comparative analysis of manually screening 1027 records versus using GPT. The analysis revealed 497 total mismatches, as detailed in Table 3. To assess the risk of false negatives, we performed a blind re-evaluation of 24 abstracts excluded by the automated system but included by human reviewers. Upon re-evaluation, all 24 were confirmed to have been correctly excluded, indicating that the discrepancies stemmed from human over-inclusion rather than model error. These findings further reinforce the reliability of the automated system in accurately identifying relevant articles when guided by clear inclusion criteria and a well-engineered prompt.

**Table 2:** Comparison of GPT and manual responses

Decision	GPT	Manual
Maybe	0	129
No	550	820
Yes	477	78

**Table 3:** Average mismatch between manual screening and GPT

Mismatch	# Articles	Average
Mismatch maybe	129	12.56%
Mismatch no	344	33.50%
Mismatch yes	24	2.34%

To quantitatively evaluate the classification performance of the GPT for screening, we computed standard metrics including precision, recall, and F1-score. Given that GPT does not generate a “maybe” label, we conservatively mapped all “maybe” decisions made by reviewers to “yes” to avoid underestimating false negatives. Under this mapping, GPT achieved a precision of 0.28, recall of 0.73, and an F1 score of 0.40 for the “yes” class, reflecting strong sensitivity and a preference for inclusion (see Table 4 for details). For the “no” class, the model showed high precision (0.91) and a solid F1 score (0.72), indicating reliable exclusion of irrelevant studies. However, the lower recall (0.59) suggests some irrelevant studies were misclassified as relevant. Table 5 presents the weighted metrics, accounting for class imbalance, which shows an overall F1-score of 0.66, precision of 0.80, and recall of 0.62, providing a balanced view of performance across both classes.

**Table 4:** Class-wise performance of GPT (human “maybe” mapped to yes)

Metric	Yes	No
Precision	0.28	0.91
Recall	0.73	0.59
F1-score	0.40	0.72

**Table 5:** Overall performance of GPT (human “maybe” mapped to yes)

Metric	Value
Precision (weighted)	0.80
Recall (weighted)	0.62
F1-score (weighted)	0.66

## Discussion

Our case study in the field of speech and language disorders demonstrates its promising results, showcasing its potential to significantly enhance and optimize the SR process by incorporating the latest advancements in the field.

### Automatic electronic databases search and data organization:

Current approaches do not support automatic article retrieval from electronic databases, necessitating manual handling of full-text articles until the abstract screening phase is complete. Our approach addresses this limitation by significantly reducing the time required to export articles. As depicted in Fig. 2, the total number of articles returned, categorized by both the searched electronic databases and the two-tier search strategy, is 34,325. The disorder-based search alone yielded over 31,000 records across various databases. This substantial volume of data would be impractical to collect manually, thus reducing the risk of missing relevant articles. Different APIs support specific data formats for exported files, impacting the time required for data fetching and organization. As detailed in table 1, we assessed the time needed to process 1000 files of various types into a formatted file for subsequent stages. Manual handling of 1000 Excel files was found to be the most efficient, requiring a minimum of 3.5 h. Extrapolating this, managing 34,325 Excel files would take approximately 120 h. In contrast, formatting 1000 unstructured text files takes over 17 h. Fig. 2 illustrated that the Embase database returned the highest number of articles, with 14,116 articles in unstructured text format. Consequently, manually organizing and fetching data from these files could extend up to 240 h, equivalent to approximately 34.29 working days based on a 7-h workday. This considerable time requirement underscores the inefficiencies of manual data processing for large volumes of unstructured text. In comparison, our automated data fetching and organization approach completes the task in just 4 s. Consequently, processing all 34,325 records takes only 137.3 s, or approximately 2.29 min. This substantial reduction in processing time highlights the efficiency of our automated method compared to traditional manual processes.

### Automatic abstract cleaning, de-duplication, and filtering:

The process of removing duplicate records is crucial to the overall efficiency and accuracy of the review process. As previously noted, many semi-automated tools lack built-in de-duplication features, necessitating manual review and removal of duplicates by users. For instance, Abstrackr requires users to perform de-duplication using separate tools before importing data into its platform This requirement is common among standalone de-duplication tools, which often need access to full documents. Conversely, some tools, such as Rayyan, EPPI-Reviewer, Covidence, and DistillerSR, provide de-duplication capabilities during the data import phase. Nevertheless, their effectiveness can be limited because abstract cleaning is usually not conducted prior to this stage, which may result in undetected duplicates. Figure 3 illustrates the significant impact of cleaning abstracts before deduplication. Utilizing standard tools for deduplication, namely Microsoft Excel, we observed a 2.62% improvement in detecting duplicate documents in Excel. In contrast, our automatic deduplication approach outperformed standard methods by identifying more duplicated data, even when using raw, uncleaned data. Time efficiency was notably improved as well; manual deduplication, which typically requires 1 to 3 h per 100 records [35], was completed by ReviewGenie in less than a second. For 34,325 records, this automation resulted in a savings of approximately 147 working days and an estimated financial saving of $24,500. Additionally, abstract cleaning significantly enhanced the performance of subsequent filtering stages. Traditional screening approaches do not support pre-text processing of abstracts to improve screening accuracy. Our two-tier filtering automated processes reduced the number of articles for abstract screening from 28,674 (after removing duplicates) to 3520 in just 2 s, representing a substantial improvement in efficiency.

### Automatic title and abstract screening:

ReviewGenie uses GPT for screening, accurately identifying 24 cases as “no” that reviewers had incorrectly labeled as “yes”. This aligns with [5], highlighting ML’s potential to reduce human error when supported by clear criteria and instructions. Although the F1-score for the “yes” class was modest (0.40), it reflects GPT’s high recall and low precision, which indicates an inclusion-focused strategy aligned with systematic review standards that prioritize minimizing false negatives. For the “no” class, high precision and a solid F1-score indicate reliable exclusion of irrelevant studies. The weighted metrics demonstrate GPT’s balanced screening performance, effectively capturing relevant studies while excluding irrelevant ones. These results highlight GPT’s potential to perform independent abstract screening, reducing workload while ensuring sufficient inclusivity to prevent missing key studies.

Furthermore, comparing the time required for manually screening 1027 records with the efficiency of ReviewGenie, it is evident that the time savings translate into substantial economic benefits and enhanced accuracy.

It is essential to automatically track the total number of tokens used, including those in queries, before submitting data to the model. Proper token management also involves filtering and cleaning records beforehand. Currently, the pricing for GPT models is based on the number of tokens processed. For instance, using a BERT-based tokenizer, the total token count for 3520 titles and abstracts amounts to 1,289,450 tokens for input, 3520 tokens for output, and 278,080 tokens for queries. According to OpenAI’s published pricing [37], GPT-3.5-turbo-instruct costs $0.002 per 1000 input tokens and $0.0015 per 1000 output tokens, resulting in an estimated total of $3.14 for the screening task. In contrast, the cost of similar work with human intervention averages around $6231. This reinforces the value of incorporating advanced AI zero learning driven tools in the systematic review process, demonstrating the potential of ReviewGenie to revolutionize systematic reviews through improved efficiency and precision.

### Limitations and future work

While ReviewGenie has demonstrated its capabilities compared to existing screening tools, it is essential to emphasize that the effectiveness of the entire process largely depends on the accuracy with which researchers specify their research scope, search keywords, databases, and inclusion and exclusion criteria. This initial input from the researcher is critical, as it guides ReviewGenie’s performance. Particularly when utilizing LLMs for screening, the definition of clear criteria for inclusion and exclusion is paramount. For research questions with less well-defined relevance criteria, LLMs may be less effective in evaluation, underscoring the importance of carefully decided criteria, even in traditional manual approaches. In this study, we utilized GPT due to its accessibility through the OpenAI API and alignment with previous research. However, relying on proprietary models can limit reproducibility, especially as models are updated over time without transparency. Future work should investigate the use of high-performing, openly available LLMs to assess their applicability in screening tasks and enable more consistent evaluation across different model architectures and prompting strategies. Furthermore, our current implementation supports a selected number of e-databases, which may limit the comprehensiveness of the search results. Future work should address these limitations by incorporating more e-databases, enhancing LLM capabilities, and extending automation to full-text retrieval and screening, ensuring a more robust and reliable systematic review process. Despite these considerations, ReviewGenie’s integration of advanced AI technologies marks a significant advancement in optimizing the systematic review process, providing substantial time and economic benefits while maintaining high accuracy levels.

## Conclusion

ReviewGenie represents a transformative leap forward in systematic review methodology. By automating and optimizing the process from article retrieval to abstract screening, it dramatically reduces time, effort, and human error, as evidenced by its ability to process thousands of articles in seconds. The integration of advanced text preprocessing and LLMs significantly enhances accuracy and efficiency, surpassing traditional methods and easing the burden on both machines and reviewers. However, the system’s effectiveness highly depends on the precision of researchers’ search parameters and criteria, which is crucial for ensuring the correct scope of the systematic review, similar to manual processes. Future improvements, such as expanding database integration and automating full-text retrieval and screening, will solidify ReviewGenie’s position as an indispensable tool for researchers, driving significant time and cost savings while setting a new standard for AI-powered research.

## Data Availability

Not applicable.

## Abbreviations

SR: Systematic review
AI: Artificial intelligence
ML: Machine learning
LLM: Large language models
e-databases: Electronic databases
